# Job satisfaction and turnover of the first group of rural-oriented tuition-waived medical students in Guangxi, China: a mixed-method study

**DOI:** 10.1186/s12875-024-02486-2

**Published:** 2024-07-04

**Authors:** Wenxu Chen, Wenjia Xu, Yanhua Chen, Chengying Xu, Jiahui Zheng, Yunfeng Zou, Bo Zhou

**Affiliations:** 1 School of Information and Management, Guangxi Medical University, Guangxi, 530021 China; 2 Liuzhou People’s Hospital, Liuzhou, Guang xi 545000 China; 3 Vanke School of Public Health, Tsinghua University, Beijing, 100084 China; 4 School of Public Health, Guangxi Medical University, Nanning, Guangxi 530021 China

**Keywords:** RTME, RTMSs, GPs, Job satisfaction, Turnover, Rural areas

## Abstract

**Background:**

In 2010, China launched a rural-oriented tuition-waived medical education (RTME) programme to train more general practitioners (GPs) to meet the needs of the rural health workforce. Motivating and maintaining GPs is an important consideration for the shortage in the rural health workforce. This study aimed to investigate job satisfaction and turnover among the first group of rural-oriented tuition-waived medical students (RTMSs) who had completed a three-year compulsory service in Guangxi, as well as the factors affecting RTMSs turnover.

**Methods:**

This study adopted a mixed-method approach. A quantitative survey of 129 RTMSs was analysed (81.6% response rate), and qualitative interviews were conducted with 30 stakeholders, including 18 RTMSs, six administrators of the County Health Bureau, and six administrators of township health centers (THCs). A t-test, chi-square test, Fisher’s exact test, and logistic regression analysis were used to examine the quantitative data, and thematic analysis was used to analyse the qualitative data.

**Results:**

Among the 129 participants, the turnover rate was high, with 103 RTMSs reporting turnover (79.84%). Interpersonal relationships scored the highest in job satisfaction (3.63 ± 0.64) among RTMSs, while working conditions were rated the lowest (2.61 ± 0.85). Marital status (odds ratio [OR] = 0.236, 95% confidence interval [95%CI] = 0.059–0.953, *P* = 0.043), only child status (OR = 8.660, 95%CI = 1.714–43.762, *P* = 0.009), and job return satisfaction (OR = 0.290, 95%CI = 0.090–0.942, *P* = 0.039) were significantly associated with turnover. Univariate analyses showed that income had a significant influence on turnover, but the relationship gone by multivariable; however it was deemed important in the qualitative study. Qualitative analysis revealed that turnover was influenced by the working atmosphere, effort-reward imbalance, professional competence, and opportunities for training and promotion.

**Conclusions:**

This study provides insights for the policymakers about the priority areas for retaining GPs in rural locations and provides reference values for the retention of GPs in other regions with a shortage of rural health workers. For RTMSs to continue providing services to rural areas, the government should improve their salaries, balance their income and workload, provide more opportunities for training and career promotion, and managers should recognise their efforts and create an optimistic working atmosphere.

**Supplementary Information:**

The online version contains supplementary material available at 10.1186/s12875-024-02486-2.

## Background

General practitioners (GPs) play an essential role in primary care. One of the most distinct challenges in primary care in China is that qualified GPs still constitute only a small proportion of physicians since the establishment of the GP system [23, 47]. People living in rural areas lack access to health-care and have poor health outcomes owing to a shortage of GPs [58]. Moreover, the difficulties in recruiting and retaining health workers caused by a reduction in health-care utilisation, as well as a lack of financial support, have led to low-quality care and a lack of trust from patients in rural areas [30, 31]. To solve this intractable problem, China launched a national programme called rural-oriented tuition-waived medical education (RTME) in 2010 [33]. The RTME programme recruits students from rural areas of central and western China and trains them as qualified GPs. After five years of tuition-free undergraduate medical education and three years of standardised residency training, they are required to work as GPs for three years in their hometown township health centers (THCs) which provide public health services and primary care services in rural China [45]. More GPs will be trained and recruited to work in THCs in rural areas through the programme, which aims to address the demand for a rural health workforce in Central and Western China [52].

The retention of working GPs in rural and remote locations remains a longstanding global challenge [17]. For instance, previous studies have shown that Australia and Thailand were experiencing shortages of primary care workers and that many GPs in these countries stayed in rural health facilities for a short period [37, 42]. In Canada, 4.6 million Canadians lack access to primary care, and in certain rural areas, approximately 75% of the population have no GPs [10]. Doctors in all specialties prefer to live and work in urban areas over rural ones [1]. According to a Vietnamese survey, 80% of physicians had a desire to work in urban areas rather than rural facilities [49]. Similarly, a mixed methods study conducted in the Philippines found that 18% of physicians preferred to stay in rural areas after compulsory services [11].

To retain GPs in rural locations, it is important to investigate factors associated with their turnover. These factors are generally understood, such as previously identified sociodemographic factors and their perception of work status [6, 14]. For example, gender, marital status, educational level, income, workload, and whether the person is an only child have all been shown to affect the retention of health professionals in rural areas [13, 24, 27, 51, 57]. Limited job opportunities for spouses and inadequate educational provisions for children are additional reasons why GPs leave rural areas [19]. In addition, a systematic review showed that job satisfaction and dissatisfaction were significant predictors of GP retention and turnover [28]. Job satisfaction can be defined as one’s attitude toward all aspects of one’s job [40]. Existing research indicates that attitudes and evaluative judgments of working status, including working environment, relationships with colleagues, and professional autonomy, play an important role in GPs retention [20, 34, 50].

Existing research into the retention of RTMSs has mainly focused on turnover intention rather than turnover [26, 56, 61]. Although turnover intention is a strong predictor and reliable proxy for actual turnover, it does not always result in turnover [36, 41]. If the RTMSs left their site after three years of compulsory services then that were considered as turnover. Additionally, most studies on RTMSs’ retention have employed a quantitative approach, which is unable to provide a deep understanding of the findings [26, 56]. Based on the conceptual framework of human capital theory, several factors have been shown to affect the turnover of employees such as income, training, and job satisfaction [32]. The research question in this study is what factors influence RTMSs’ turnover in the RTME programme? Our study used a mixed-method approach to comprehensively analyse the results. In this study, we aimed to explore the impact of sociodemographic factors and job satisfaction components on actual turnover among GPs who had completed a three-year compulsory service in the RTME programme to identify the most influential factors. This study provides insights for the policymakers about the priority areas for retaining GPs in rural locations and provides consolidated evidence for improving the availability of the rural health workforce.

## Methods

### Study design

We used a sequential explanatory mixed-method study design because it enabled a better understanding of the factors related to job satisfaction and turnover of RTMSs than can be achieved using an individual quantitative or qualitative approach. A quantitative survey was followed by qualitative interviews. Interviews were coded using quantitative data as an analytic framework, and the qualitative results were used to explain and enrich the quantitative findings.

#### Study participants

This study was conducted from November to December 2021 in Guangxi, Western China, one of the provinces that has been implementing the RTME programme since 2010. A purposive sampling method was used to recruit the RTMSs. The inclusion criteria were as follows: (1) graduated on time, (2) completed three years of standardised residency training and three years of compulsory services, and (3) provided informed consent to participate in the study. In 2021, the first group of RTMSs in Guangxi completed the compulsory service. No one failed to complete the 3 years of service, and 158 RTMSs were invited to complete the web-based questionnaire online, with a response rate of 81.6% and 129 valid survey questionnaires. Additionally, we recruited 18 RTMSs (including both retention and turnover RTMSs), six administrators of the County Health Bureau, and six administrators of THCs to participate in telephone interviews. Eighteen RTMSs were selected purposively. The administrators of the Health Bureau and THCs came from six different counties in Guangxi: Mengshan, Mashan, Gongcheng, Sanjiang, Gangbei, and Wuming. The 30 stakeholders were interviewed by two PhD researchers (WC and WX) who were familiar with the research design and background and were trained in qualitative research methods. One researcher (WX) conducted the interviews and the other (WC) recorded the responses.

This study was approved by the Ethics Review Board of the School of Public Health at Guangxi Medical University. All participants provided informed consent before participating the study.

#### Measurement and data collection

The questionnaire included three sections: sociodemographic characteristics, job satisfaction scale, and turnover. Sociodemographic variables included age (≤ 30, > 30), gender (female, male), education level (bachelor, master), marital status (unmarried, married), only child or not (yes, no), daily working hours (< 6, 6–8, > 8), and monthly income in CNY (≤ 3000, 3001–5000, 5001–7000, > 7000). Data on job satisfaction were gathered using self-designed questionnaires with 26 items that evaluated the five aspects of the RTMSs, including work itself (seven items), job return (six items), working conditions (five items), professional growth (five items), and interpersonal relationships (three items) (Appendix Table S1 and Table S2). Items were developed based on existing questionnaires, such as the Minnesota Satisfaction Questionnaire (MSQ) and the Job Satisfaction Survey (JSS). Satisfaction with the RTMSs was reported using a five-point Likert scale ranging from 1 (very dissatisfied) to 5 (very satisfied). Using a five-point Likert scale, RTMSs with a score of less than three were classified as dissatisfied in the regression. The Cronbach’s alphas for the five subscales were 0.903, 0.925, 0.891, 0.936, and 0.795, respectively, indicating high reliability. A single item was developed to measure actual turnover by asking, “Did you continue working in the same THC you were posted after a three-year mandatory service?”.

We conducted semi-structured telephone interviews to better understand the working status, job satisfaction, and retention concerns of RTMSs in rural locations. In addition, we conducted telephone interviews to determine what actions should be taken for RTMSs’ retention from the administrators’ perspectives (Appendix Table S3). Each interview lasted for 30 to 60 min using a semi-structured interview guide, and all interviews were audio-recorded and transcribed verbatim.

#### Data analysis

Descriptive statistics were used to describe the sociodemographic characteristics, job satisfaction, and turnover rates of RTMSs. We obtained frequency (n) and percentage (%) statistics to show the sociodemographic factors and turnover rate, in addition to the mean ± standard deviation (SD), to report the levels of job satisfaction. Differences in turnover according to sociodemographic characteristics and job satisfaction were analysed using t-tests and chi-square tests. Fisher’s exact test was used as an alternative to the chi-square test in cases where the expected frequency fell below five. Logistic regression analysis was used to examine the factors influencing turnover. We used a *P* value of 0.25 as a cut-off point for the stopping rule to select predictors in the multivariable logistic regression analysis [2]. Odds ratios (ORs) with corresponding 95% confidence intervals (CI) are reported. All analyses were performed using Stata 17, and statistical significance was set at *P* < 0.05.

A confirmatory factor analysis (CFA) was conducted to verify the measurement model of the job satisfaction constructs. The goodness-of-fit of the models was evaluated using a set of indices, including the chi-square value/degrees of freedom (χ^2^/df), root mean square error of approximation (RMSEA), goodness-of-fit index, adjusted goodness-of-fit index (AGFI), comparative fit index (CFI), Tucker-Lewis index (TLI), and incremental fit index (IFI). The model fit was considered acceptable if χ^2^/df < 3, RMSEA < 0.08, GFI > 0.90, AGFI > 0.90, CFI > 0.90, TLI > 0.90, and IFI > 0.90 [9, 12]. The CFA was performed using AMOS 25.0; further information and CFA results are included in Appendix Table S4 and Figure S1.

For the semi-structured interviews, qualitative data were analysed using thematic analysis with a combination of deductive and inductive processes. Subsequently, we organised and categorised the codes into different themes/subthemes and generated a final coding framework by two researchers (WC and WX), considering inter-rater reliability. Interview transcripts were read repeatedly and open-coded. Second, the concepts were grouped into 2nd order themes based on their relationships, similarities, and differences. Finally, these themes were further abstracted as aggregate dimensions [41, 60]. Theoretical coding was performed using NVivo 11.0 plus.

## Results

### Quantitative results

#### Characteristics of participants

Table 1 presents the sociodemographic characteristics of the RTMSs. The mean age of the RTMSs was 30.59 years (range, 29–34 years). Among the 129 RTMSs, 65 (50.4%) were male, and 103 (96.1%) had a bachelor’s degree. Most RTMSs (92.2%) were not the only child, and approximately two-thirds were married (67.4%). Most RTMSs (over 70%) worked for more than eight hours per day. Almost half earned 3001–5000 CNY per month (58.9%), 24% earned 5001–7000 CNY per month, and only 8.5% earned over 7000 CNY per month. The average wage rate of urban non-private sector employees in Guangxi in 2021 was 7348 CNY per month [3]. Overall, 103 of the 129 (79.84%) RTMSs in the present study reported that they had left THCs, suggesting a high turnover rate. Among the 129 participants, 103 (79.84%) reported that they had left THCs and the overall retention rate was 20.16%, suggesting a high turnover rate.

**Table 1:** Sociodemographic characteristics of RTMSs (*N* = 129)

Variable	N (%)	Turnover		χ^2^/t	*P* value
		Yes	No		
		N (%) or Mean ± SD	N (%) or Mean ± SD		
Gender				4.625	0.032
Male	65 (50.4)	47 (72.3)	18 (27.7)		
Female	64 (49.6)	56 (87.5)	8 (12.5)		
Age (years)		30.50 ± 0.96	30.92 ± 1.35	1.484	0.148
≤ 30	71 (55.0)	57(80.3)	14(19.7)		
> 30	58 (45.0)	46(79.3)	12(20.7)		
Education level				1.313	0.582*
Bachelor	124 (96.1)	98 (79.0)	26 (21.0)		
Master	5 (3.9)	5 (100)	0 (0)		
Marital status				6.552	0.010
Unmarried	42 (32.6)	39 (92.9)	3 (7.1)		
Married	87 (67.4)	64 (73.6)	23 (26.4)		
Only child or not				6.000	0.028*
Yes	10 (7.8)	5 (50.0)	5 (50.0)		
No	119 (92.2)	98 (82.4)	21 (17.6)		
Daily working hours				0.057	0.812
≤ 8 h	27 (20.9)	22 (81.5)	5 (18.5)		
> 8 h	102 (79.1)	81 (79.4)	21 (20.6)		
Monthly income (CNY)				12.376	0.008*
≤ 3000	11 (8.5)	11 (100)	0 (0)		
3001–5000	76 (58.9)	64 (84.2)	12 (15.8)		
5001–7000	31 (24.0)	23 (74.2)	8 (25.8)		
> 7000	11 (8.5)	5 (45.5)	6 (54.5)		

^*^Fisher’s exact test

#### RTMSs’ job satisfaction

Table 2 presents the job satisfaction of the RTMSs. The subscale with which RTMSs were most satisfied was interpersonal relationships (81.4%), followed by work itself (55.8%), job return (31.0%), professional growth (26.4%), and working conditions (22.5%).

**Table 2:** Job satisfaction of RTMSs (*N* = 129)

Variable	N (%) or Mean ± SD	Turnover		χ^2^	*P* value
		Yes	No		
		N (%)	N (%)		
Work itself	3.30 ± 0.92			3.935	0.047
Satisfied	72 (55.8)	53 (73.6)	19 (26.4)		
Dissatisfied	57 (44.2)	50 (87.7)	7 (12.3)		
Job return	2.71 ± 0.87			10.839	0.001
Satisfied	40 (31.0)	25 (62.5)	15 (37.5)		
Dissatisfied	89 (69.0)	78 (87.6)	11 (12.4)		
Working conditions	2.61 ± 0.85			1.284	0.257
Satisfied	29 (22.5)	21 (72.4)	8 (27.6)		
Dissatisfied	100 (77.5)	82 (82.0)	18 (18.0)		
Professional growth	2.62 ± 1.00			2.458	0.117
Satisfied	34 (26.4)	24 (70.6)	10 (29.4)		
Dissatisfied	95 (73.6)	79 (83.2)	16 (16.8)		
Interpersonal relationships	3.63 ± 0.64			0.223	0.782*
Satisfied	105 (81.4)	83 (79.0)	22 (21.0)		
Dissatisfied	24 (18.6)	20 (83.3)	4 (16.7)		
Overall job satisfaction	2.96 ± 0.69			6.400	0.011
Satisfied	56 (43.4)	39 (69.6)	17 (30.4)		
Dissatisfied	73 (56.6)	64 (87.7)	9 (12.3)		

^*^Fisher’s exact test

#### Univariate analysis of turnover

There was a statistically significant difference in turnover according to gender, marital status, only child, income, work itself satisfaction, and job return satisfaction (*P* < 0 0.05). The RTMSs who were female, married, and non-only child were more likely to leave their positions, and the RTMSs with lower incomes were more likely to leave their positions. The RTMSs who were dissatisfied with work itself and job return were more likely to leave their positions than those who were satisfied (Table 2).

#### Logistic regression analysis of turnover

Table 3 presents the ORs and 95%CI calculated using the multivariable logistic regression models. We found that marital status (OR = 0.236, 95%CI = 0.059–0.953, *P* = 0.043), only child status (OR = 8.660, 95%CI = 1.714–43.762, *P* = 0.009), and job return satisfaction (OR = 0.290, 95% CI = 0.090–0.942, *P* = 0.039) were associated with turnover.

**Table 3:** Logistic regression analysis on turnover of RTMSs (*N* = 129)

Variable	OR^*^	*P* value	95%CI
Gender			
Male	Ref		
Female	0.722	0.577	(0.230–2.268)
Age (years)	0.720	0.198	(0.437–1.187)
Marital status			
Unmarried	Ref		
Married	0.236	0.043	(0.059–0.953)
Only child or not			
Yes	Ref		
No	8.660	0.009	(1.714–43.762)
Monthly income (CNY)	0.506	0.062	(0.248–1.034)
Work itself			
Dissatisfied	Ref		
Satisfied	1.288	0.700	(0.355–4.670)
Job return			
Dissatisfied	Ref		
Satisfied	0.290	0.039	(0.090–0.942)
Professional growth			
Dissatisfied	Ref		
Satisfied	0.758	0.638	(0.239–2.405)

^*^Adjusted odds ratio

#### Qualitative results

Table 4 presents the aggregate dimensions, themes, and concepts of the interview codes with the RTMSs. The major aggregate dimensions were job demands, working conditions, career prospects, and personal factors.

**Table 4:** Aggregate dimensions, themes, and concepts of codes of the interview with RTMSs

Aggregate Dimensions	2nd Order Themes	1st Order Concepts
Job demands	Workload	“In fact, the hospital where I work is busy, and the workload is quite heavy” (RTMS who stayed in the position) “The workload is heavy, and I work more than ten hours per day. Almost everyone who works here has a lot of work to do” (RTMS who left the position)
	Job stress	“Pressures come from two major sources: hospital performance and disease prevention and control” (RTMS who stayed in the position) “There is limited guidance regarding clinical practice at THCs, which makes me feel stressed” (RTMS who left the position)
Working conditions	Team cohesion	“I enjoy my job and get along well with my managers and colleagues” (RTMS who stayed in the position) “I have a good relationship with colleagues, and my managers care for me” (RTMS who left the position)
	Working environment	“The issues of working in THCs are the shortage of medical equipment and drug” (RTMS who stayed in the position) “There are some difficulties of working in THCs, such as the poor working environment. Additionally, it takes five or six hours to drive from THCs to counties, so I have to engage in long-distance commuting” (RTMS who stayed in the position) “The biggest challenge is the shortage of staff, and there are less than 30 healthcare workers at this health facility” (RTMS who left the position)
	Working atmosphere	“The working atmosphere at THCs was not good, and my colleagues did not have a passion for studying” (RTMS who left the position) “The working atmosphere was another reason for turnover” (RTMS who left the position)
Career prospects	Promotion	“I had opportunities for promotion in THCs, so I stayed in my position” (RTMS who stayed in the position) “I stayed in my position because of managers” support, and I got a promotion during working in THCs” (RTMS who stayed in the position)
	Opportunities for CME and training	“I have few opportunities for CME and training” (RTMS who left the position) “I left my position because there were no opportunities for training.” (RTMS who left the position)
	Income	“The dominant reason for turnover was the poor remuneration” (RTMS who left the position) “The salary was lower than expected, which was the reason for turnover” (RTMS who left the position)
	Effort-reward imbalance	“There was an imbalance between effort and reward, and I perceived a gap between ideals and realities at work” (RTMS who left the position) “Another reason for turnover was the effort-reward imbalance” (RTMS who left the position)
	Professional competence	“I left my position because I want to improve my professional competence, and I intended to pursue a PhD degree” (RTMS who left the position) “I left my position because I felt incompetent and wanted to improve myself” (RTMS who left the position)
Personal factors	Distance from home	“I stayed in my position because it will be difficult to spend time with my family members if the health facility is far from home” (RTMS who stayed in the position) “I left my position because the distance from home separated me from my husband.” (RTMS who left the position)
	Sense of accomplishment	“In fact, it gives me a sense of accomplishment when providing needed services to rural patients.” (RTMS who stayed in the position) “I learned a lot in college but could not demonstrate my competence in rural areas.” (RTMS who left the position)

#### Job demands

Some RTMSs faced high job demands in rural areas. They said that their workload was heavy, working more than ten hours a day, and that the RTMSs employed at THCs were overloaded with work. Beyond a heavy workload, they also experienced stress owing to job performance and disease prevention and control work as well as limited clinical practice guidelines from THCs.

#### Working conditions

Most RTMSs believed that they experienced high levels of team cohesion and poor working environments at THCs. They got along with their managers and colleagues. However, the main challenges of working in THCs were a poor working environment, including inadequate medical equipment and drugs, a shortage of healthcare workers, and the long distances they had to commute from THCs and counties. Additionally, some RTMSs expressed dissatisfaction with their working atmosphere in rural workplaces. For example, their colleagues showed no enthusiasm for continuing medical education (CME).

#### Career prospects

Attitudes toward career prospects influenced RTMSs’ decisions to work in rural areas. For instance, some RTMSs remained in rural areas because they were promoted at THCs. Some RTMSs departed from rural areas because of limited opportunities for CME and training in THCs. Some RTMSs mentioned that their salaries were lower than expected and poor remuneration was the primary cause of turnover. Some RTMSs perceived a gap between ideals and realities at work, and they left because of the effort-reward imbalance. Furthermore, some RTMSs left because they wanted an increase in their competency. For instance, after leaving the THCs, one RTMSs wanted to pursue a PhD.

#### Personal factors

Personal considerations, such as a sense of self-accomplishment and distance from home, influenced their decision to leave or remain in rural areas. Regarding distance from home, some RTMSs remained in their positions because they wanted to spend time with their families, whereas others left because their family members lived elsewhere. The RTMSs also noted a sense of accomplishment in providing the services needed for rural patients as a factor in their ongoing employment in THCs.

Table 5 presents the aggregate dimensions, themes, and codes of the interviews with administrators at the County Health Bureau and THCs. The major aggregate dimensions identified were financial incentives, non-financial incentives, and success in the RTME programme.

**Table 5:** Aggregate dimensions, themes, and concepts of codes of the interview with administrators

Aggregate Dimensions	2nd Order Themes	1st Order Concepts
Financial incentives	Salary and welfare	“It is necessary to raise RTMSs’ wages” “It is critical to increasing the salary and welfare of RTMSs”
	Financial investment	“The large increase in financial investment is beneficial for RTMSs’ retention”
Non-financial incentives	Working environment	“The working environment should be improved to the greatest extent possible”
	Professional fulfilment	“We need to pay more attention to RTMSs, and give them an opportunity to demonstrate their competence”
	Opportunities for career development	“It is important to provide RTMSs with more opportunities for training” “Providing opportunities for promotion is beneficial for RTMSs’ retention”
Success in RTME programme	GPs recruitment	“This programme alleviates the shortage of GPs and, to some extent, addresses the problem of recruitment”
	Healthcare services	“The RTME programme can improve the efficiency and quality of healthcare services”

#### Financial incentives

Most administrators believed that financial incentives provided incentives for RTMSs to continue serving rural areas. Thus, financial investment was needed not only for RTMSs but also for the RTME programme.

#### Non-financial incentives

In addition to financial factors, administrators noted non-financial incentives as incentives for RTMSs retention. These included improving their working environment, enhancing their sense of fulfilment, and offering them more career pathways.

#### Success in the RTME programme

All administrators acknowledged that more GPs provided high-quality health care in rural areas as a result of the nationwide RTME programme. The RTME programme successfully alleviated the shortage of GPs and addressed the recruitment problem in Central and Western China.

## Discussion

To the best of our knowledge, this is the first study to investigate job satisfaction and turnover among RTMSs who have completed a three-year compulsory service in western China. This study maintains a stable health workforce in rural China and provides concrete suggestions for the retention of GPs in other regions experiencing a shortage of rural health workers. We explored the factors affecting RTMSs turnover by integrating quantitative and qualitative data, enabling a comprehensive analysis of the findings. Quantitative findings showed that interpersonal relationships scored the highest in job satisfaction among RTMSs, while working conditions were rated the lowest. Besides, marital status, only child status, and job return satisfaction were associated with turnover. The qualitative analysis of interviews of 18 RTMSs identified four themes, including job demands, working conditions, career prospects and personal factors; results from interviews with 12 administrators revealed three themes such as financial incentives, non-financial incentives and success in RTME programme. Both quantitative and qualitative elements validated the findings of this study.

The univariate analysis revealed that female RTMSs were more likely to experience turnover. This finding is inconsistent with a study conducted in England, which found that male GPs were more likely to leave their jobs than female GPs [8]. A possible explanation for this gender difference is that female GPs in rural China may be more affected by work-family conflict, and their employment decisions are more influenced by family commitments and responsibilities [7, 39]. Additionally, our study found that the working atmosphere was a critical factor influencing RTMSs’ turnover. This finding contradicts a report from Australia in which GPs had a good working atmosphere in rural areas and identified rural practices as opportunities to improve their skills [21]. One possible explanation is that most primary healthcare workers at THCs in China had low levels of educational attainment and showed no enthusiasm for CME, which failed to generate healthy competition among the RTMSs and prevented them from developing their abilities [18]. To retain RTMSs, administrators should endeavour to foster a positive working atmosphere and increase their enthusiasm for work. In addition, the RTMSs reported higher turnover rate than overall doctors [46]. One possible explanation for this is that the service RTMSs provided was compulsory and they did not have a good understanding of the policy of the RTME programme when they graduated from high school [26].

In this study, the sampled RTMSs were mostly satisfied with their interpersonal relationships while working in rural areas. This was confirmed by the qualitative findings that they got along with their managers and colleagues at the THCs. This finding is consistent with a report from Germany in which GPs generally reported high levels of satisfaction with their interpersonal relationships [43]. This is a positive signal because being recognised by managers and building harmonious relationships with colleges could motivate GPs to stay in rural areas [44]. However, we also observed that the RTMSs were subject to poor working conditions at the THCs. This finding is in line with that of an Indian study that indicated that GPs expressed dissatisfaction with their working conditions [15]. Poor working conditions, including an insufficient supply of medical equipment and drugs, a lack of medical staff, and the long distance they had to commute from THCs and counties, were identified as challenges in our qualitative analysis. Targeted actions are essential to improve RTMSs’ working conditions satisfaction. In particular, the availability of medical equipment and drugs is vital for healthcare delivery at THCs [4].

Our study found that those who were unmarried and not the only child were more likely to experience turnover. In terms of marital status, our findings are in line with those of a report from Tanzania, where unmarried primary healthcare workers were more likely to leave their jobs than those who were married [35]. One possible explanation is that unmarried RTMSs have fewer family responsibilities, thus they do not have to consider relocating their family members when leaving their current positions [35, 48]. The RTMSs who were not the only child were more likely to leave rural areas than those who were the only child. This may be because those who are the only child are the main care-giving providers for their ageing parents, forcing them to remain in rural areas to fulfil their obligations [5, 59]. In addition, our qualitative analysis revealed that RTMSs prefer to leave rural areas because their family members live in other places. This finding is consistent with that of a survey in Australia, where sufficient time to stay with family members influenced GPs’ decisions to work in rural areas [55]. To retain RTMSs, employment opportunities for spouses should be provided, and early childhood education should be developed in rural areas.

In the present study, RTMSs who were dissatisfied with their job return were more likely to experience turnover. The qualitative analysis confirmed that RTMSs complained of poor remuneration and an effort-reward imbalance when working in rural areas. This finding is consistent with previous studies which indicated that GPs’ low salaries were disproportionate to their heavy workload and stress, which may have affected their turnover [16, 54]. Moreover, our study found that income and work itself satisfaction significantly influenced turnover in univariate analyses. To retain RTMSs, the government should raise wages and balance income and workload. The government should offer favourable policies to motivate RTMSs such as a performance‐based pay system, housing allowance and transportation allowances, as the salaries of GPs are paid by the local government’s treasury [22, 38]. In addition, managers should help RTMSs fulfil the value of their efforts.

Our qualitative findings indicated that professional competence and opportunities for CME and training were also key factors that influenced RTMSs’ turnover. This finding is in line with previous studies which found that limited accessibility to CME and training hindered GPs from improving professional competency and further led to turnover [25, 31, 54]. To retain RTMSs, the government should provide them with more opportunities for CME and general practice on-the-job training program. The training program covers the basics knowledge of general practice and primary medical care, which will enhance trainees’ knowledge and skills [53].. Furthermore, promotion opportunities were found to directly affect RTMSs’ turnover. This is supported by a previous study showing that managers’ support, such as promotions, could enhance GPs’ job performance and increase their commitment to rural practices [29]. According to the administrators surveyed in this study, even if the national policy is successful in alleviating the shortage of GPs and providing high-quality care to rural communities, financial investments and non-financial efforts should be considered in the future. For instance, to improve the RTME programme, urgent action is needed to create a better working atmosphere and provide RTMSs with more opportunities for career development.

This study had several limitations. First, because this study employed a retrospective self-report approach to collect data, recall bias was unavoidable. Second, self-reports were used to collect data from the RTMSs, which might have limited the reliability of some responses. For example, the respondents were inclined to report high satisfaction ratings. Third, although the sample covers all RTMSs that have completed a three-year compulsory service in Guangxi province, the extrapolation of conclusions to the national level could be challenging. Finally, owing to the cross-sectional nature of the quantitative design of this study, the association between sociodemographic characteristics, job satisfaction, and turnover could not be concluded as a causal relationship.

## Conclusions

The findings of this study may be considered by healthcare administrations in the hope of informing recruitment and retention policies, as well as providing reference values for the retention of GPs in other regions with a shortage of rural health workers. Strategies are recommended to achieve the goal of retaining RTMSs, including raising wages, balancing income and workload, improving working conditions, particularly supplying adequate medical equipment and drugs, creating an optimistic working atmosphere, and providing more opportunities for CME and career development.

### Supplementary Information

Supplementary Material 1.

## Data Availability

The datasets used and/or analysed during the current study are available from the corresponding author on reasonable request.

## Abbreviations

AGFI: Adjusted goodness of fit index
CFA: Confirmatory factor analysis
CFI: Comparative fit index
CI: Confidence intervals
CME: Continuing medical education
CNY: Chinese Yuan
GFI: Goodness of fit index
GPs: General practitioners
IFI: Incremental fit index
ORs: Odds ratios
RMSEA: Root mean square error of approximation
RTME: Rural-oriented tuition-waived medical education
RTMSs: Rural-oriented tuition-waived medical students
SD: Standard deviation
THCs: Township health centers
TLI: Tucker-Lewis index
χ^2^/df: Chi-square/degrees of freedom

## References

[1] Barreto T, Jetty A, Eden AR, Petterson S, Bazemore A, Peterson LE (2021). Distribution of physician specialties by rurality. J Rural Health.

[2] Bendel RB, Afifi AA (1977). Comparison of stopping rules in forward “stepwise” regression. J Am Stat Assoc.

[3] Bureau of Statistics of Guangxi. The average wage rate of urban non-private sector employees in Guangxi in 2021. 2022. Available: http://tjj.gxzf.gov.cn/tjsj/xwfb/tjxx_sjfb/t11976739.shtml?eqid=d5835440000217c6000000066445eece. Accessed 12 Feb 2024.

[4] Chen G, Sang L, Rong J, Yan H, Liu H, Cheng J, Wang L, Ding H, Chen R (2021). Current status and related factors of turnover intention of primary medical staff in Anhui Province, China: a cross-sectional study. Hum Resour Health.

[5] Chen X, Zhuoga C, Deng Z (2021). Adaptations to the one-child policy: Chinese young adults' attitudes toward elder care and living arrangement after marriage. Front Psychol.

[6] Chen YQ, You YW, Zhang Q, Wang YD, Dai T (2022). Systematic evaluation of influencing factors for Chinese rural doctors' job satisfaction and turnover intention: based on the two-factor theory. Eur Rev Med Pharmacol Sci.

[7] Cooper CL, Rout U, Faragher B. Mental health, job satisfaction, and job stress among general practitioners. In: Managerial, occupational and organizational stress research. London: Routledge; 2018. 10.1136/bmj.298.6670.366 PMC1835734 2493939

[8] Dale J, Potter R, Owen K, Parsons N, Realpe A, Leach J (2015). Retaining the general practitioner workforce in England: what matters to GPs? A cross-sectional study. BMC Fam Pract.

[9] Dou H, Zhao Y, Chen Y, Zhao Q, Xiao B, Wang Y, Zhang Y, Chen Z, Guo J, Tao L (2018). Brief adult respiratory system health status scale-community version (BARSHSS-CV): developing and evaluating the reliability and validity. BMC Health Serv Res.

[10] Fleming P, Sinnot ML. Rural physician supply and retention: factors in the Canadian context. 2018.

[11] Flores ELL, Manahan EMR, Lacanilao MPB, Ladaw IMBT, Mallillin MMB, Mappatao NTQ, Leonardia JA, Pepito VCF (2021). Factors affecting retention in the Philippine National Rural Physician Deployment Program from 2012 to 2019: a mixed methods study. BMC Health Serv Res.

[12] Gambashidze N, Hammer A, Manser T (2019). Psychometric properties of the Georgian version of hospital survey on patient safety culture: a cross-sectional study. BMJ Open.

[13] Gan Y, Gong Y, Chen Y, Cao S, Li L, Zhou Y, Herath C, Li W, Song X, Li J (2018). Turnover intention and related factors among general practitioners in Hubei, China: a cross-sectional study. BMC Fam Pract.

[14] Gan Y, Jiang H, Li L, Yang Y, Wang C, Liu J, Yang T, Zheng Y, Zhu Y, Sampson O (2020). A national survey of turnover intention among general practitioners in China. Int J Health Plann Manag.

[15] Gu J, Zhen T, Song Y, Xu L (2019). Job satisfaction of certified primary care physicians in rural Shandong Province, China: a cross-sectional study. BMC Health Serv Res.

[16] HäMMIG O (2018). Explaining burnout and the intention to leave the profession among health professionals–a cross-sectional study in a hospital setting in Switzerland. BMC Health Serv Res.

[17] Iglehart JK (2018). The challenging quest to improve rural health care. N Eng J Med.

[18] Jin Y, Wang H, Wang D, Yuan B (2019). Job satisfaction of the primary healthcare providers with expanded roles in the context of health service integration in rural China: a cross-sectional mixed methods study. Hum Resour Health.

[19] Jolicoeur J, Demiglio L, Kin LNR, Orrantia E (2022). Why they leave: small town rural realities of northern physician turnover. Can J Rural Med.

[20] Kim H, Stoner M (2008). Burnout and turnover intention among social workers: effects of role stress, job autonomy and social support. Adm Soc Work.

[21] Laurence CO, Williamson V, Sumner KE, Fleming J (2010). "Latte rural": the tangible and intangible factors important in the choice of a rural practice by recent GP graduates. Rural Remote Health.

[22] Li H, Liu K, Gu J, Zhang Y, Qiao Y, Sun X (2017). The development and impact of primary health care in China from 1949 to 2015: a focused review. Int J Health Plann Manage.

[23] Li X, Krumholz HM, Yip W, Cheng KK, de Maeseneer J, Meng Q, Mossialos E, Li C, Lu J, Su M (2020). Quality of primary health care in China: challenges and recommendations. Lancet.

[24] Lin T, Li Y, Li Y, Guo W, Guo X, Tang C (2023). Individual-and institution-level predictors of the turnover intention of medical staff among rural primary medical institutions in Xinjiang Production and Construction Corps, China: a cross-sectional multi-level analysis. Front Psychol.

[25] Liu J, Mao Y (2020). Continuing medical education and work commitment among rural healthcare workers: a cross-sectional study in 11 western provinces in China. BMJ Open.

[26] Liu J, Zhang K, Mao Y (2018). Attitude towards working in rural areas: a cross-sectional survey of rural-oriented tuition-waived medical students in Shaanxi, China. BMC Med Educ.

[27] Liu J, Zhu B, Wu J, Mao Y (2019). Job satisfaction, work stress, and turnover intentions among rural health workers: a cross-sectional study in 11 western provinces of China. BMC Fam Pract.

[28] Long L, Moore D, Robinson S, Sansom A, Aylward A, Fletcher E, Welsman J, Dean SG, Campbell JL, Anderson R (2020). Understanding why primary care doctors leave direct patient care: a systematic review of qualitative research. BMJ Open.

[29] Mandal A, Phillips S (2022). To stay or not to stay: the role of sense of belonging in the retention of physicians in rural areas. Int J Circumpolar Health.

[30] Mathers N, Huang YC. The future of general practice in China: from ‘barefoot doctors’ to GPs?. Br J Gen Pract. 2014. 10.3399/bjgp14X679933 PMC4031987 24868042

[31] Meng Q, Yuan J, Jing L, Zhang J (2009). Mobility of primary health care workers in China. Hum Resour Health.

[32] Mudor H (2011). Conceptual framework on the relationship between human resource management practices, job satisfaction, and turnover. J Econ Behav Stud.

[33] National Development and Reform Commission. Proclamation on implementing rural-oriented tuition-waived medical education programme. 2010. Available: https://www.ndrc.gov.cn/fzggw/jgsj/shs/sjdt/201006/t20100604_1121988.html?code=&state=123. Accessed 30 Jul 2022.

[34] Nazir S, Shafi A, Qun W, Nazir N, Tran QD (2016). Influence of organizational rewards on organizational commitment and turnover intentions. Employ Relat.

[35] Ngilangwa DP, Mgomella GS (2018). Factors associated with retention of community health workers in maternal, newborn and child health programme in Simiyu Region, Tanzania. Afr J Prim Health Care Fam Med.

[36] Oh S, Kim H (2019). Turnover intention and its related factors of employed doctors in Korea. Int J Environ Res Public Health.

[37] Pagaiya N, Kongkam L, Sriratana S (2015). Rural retention of doctors graduating from the rural medical education project to increase rural doctors in Thailand: a cohort study. Hum Resour Health.

[38] Qing Y, Hu G, Chen Q, Peng H, Li K, Wei J, Yi Y (2015). Factors that influence the choice to work in rural township health centers among 4,669 clinical medical students from five medical universities in Guangxi, China. J Educ Eval Health Prof.

[39] Ravaghi H, Taati E, Abdi Z, Meshkini A, Sarvarizadeh S (2015). Factors influencing the geographic distribution of physicians in Iran: a qualitative study. Rural Remote Health.

[40] Ravari A, Mirzaei T, Kazemi M, Jamalizadeh A (2012). Job satisfaction as a multidimensional concept: a systematic review study. J Occup Health Epidemiol.

[41] Ruotsalainen S, Jantunen S, Sinervo T (2020). Which factors are related to Finnish home care workers’ job satisfaction, stress, psychological distress and perceived quality of care?-a mixed method study. BMC Health Serv Res.

[42] Russell DJ, McGrail MR, Humphreys JS (2017). Determinants of rural Australian primary health care worker retention: a synthesis of key evidence and implications for policymaking. Aust J Rural Health.

[43] Sang L, Chen R (2022). Incentive preferences and its related factors among primary medical staff in Anhui Province, China: a cross-sectional study. Front Public Health.

[44] Seangrung R, Chuangchum P (2017). Factors affecting the rural retention of medical graduates in lower northern Thailand. J Med Assoc Thai.

[45] Shen Y, Huang X, Li H, Chen E, Kong Y, Yu J, Liu X, Mobarak SA, Zuo Y (2021). Early outcomes of a rural-oriented physician education programme against rural physician shortages in Guangxi province: a prospective cohort study. BMJ Open.

[46] Shi K, Wang Y, Sun Z, Zhao J, Xiang F, Chen Z, Sun W, Zheng Y (2024). Turnover behavior and intention among dentists and medical doctors: a cross-sectional study in China. BMC Oral Health.

[47] Shu Z, Wang Z, Chen R, Li M, Lou J, Huang X, Wu J, Jing L (2017). Allocation and development of the general practitioner workforce in China from 2012 to 2015: a literature review. Lancet.

[48] Tai TWC, Bame SI, Robinson CD (1998). Review of nursing turnover research, 1977–1996. Soc Sci Med.

[49] Vujicic M, Shengelia B, Alfano M, Thu HB (2011). Physician shortages in rural Vietnam: using a labor market approach to inform policy. Soc Sci Med.

[50] Wang H, Jin Y, Wang D, Zhao S, Sang X, Yuan B (2020). Job satisfaction, burnout, and turnover intention among primary care providers in rural China: results from structural equation modeling. BMC Fam Pract.

[51] Wang J, Chen Y, Zhang H, Zhang J. Turnover intention and continuing professional development of rural doctors from targeted admission medical education program in China: a cross-sectional study in the post-COVID-19 era. 2023. 10.1186/s12875-024-02637-5 PMC11533297 39497040

[52] Wang L, Yang Y, Zhu J, Xie H, Jiang C, Zhang C, Li J, Huang F (2019). Professional identity and mental health of rural-oriented tuition-waived medical students in Anhui Province, China. BMC Med Educ.

[53] Wen R, Ying L, Yan Q, Jingjing R (2014). Development of general practice education and training in China. Chin Med J.

[54] Wen T, Zhang Y, Wang X, Tang G (2018). Factors influencing turnover intention among primary care doctors: a cross-sectional study in Chongqing, China. Hum Resour Health.

[55] Wieland L, Ayton J, Abernethy G (2021). Retention of general practitioners in remote areas of Canada and Australia: a meta-aggregation of qualitative research. Aust J Rural Health.

[56] Yan W, Gao X, Wang W, Zhou Z, Zou C, Lu Z (2022). Job satisfaction of graduates of rural oriented medical students training project in Jiangsu Province, China: a cross-sectional study. BMC Med Educ.

[57] Yan W, Sun G (2022). Income, workload, and any other factors associated with anticipated retention of rural doctors?. Prim Health Care Res Dev.

[58] Yang L, Cheng J (2022). Analysis of inequality in the distribution of general practitioners in China: evidence from 2012 to 2018. Prim Health Care Res Dev.

[59] Zhan HJ (2002). Chinese caregiving burden and the future burden of elder care in life-course perspective. Int J Aging Hum Dev.

[60] Zhao X, Wang H, Li J, Yuan B (2020). Training primary healthcare workers in China’s township hospitals: a mixed methods study. BMC Fam Pract.

[61] Zou C, Liao XY, Spicer J, Hayhoe B (2020). Ten years’ GP training in China: progress and challenges. Br J Gen Pract.

