# Supplementary material for: Job satisfaction and turnover of the first group of rural-oriented tuition-waived medical students in Guangxi, China: a mixed-method study
Source: BMC Prim Care. 2024 Jul 4;25:237. doi: 10.1186/s12875-024-02486-2 (PMC11225299; doi:10.1186/s12875-024-02486-2)
Supplement: Supplementary file 1 — Supplementary Material 1. [file 12875_2024_2486_MOESM1_ESM.docx]

| Table S1 Job satisfaction scale | | | | | |
| --- | --- | --- | --- | --- | --- |
| For each statement, please circle the number to indicate your degree of agreement | | | | | |
| Item | Strongly Disagree | Disagree | Don’t Know | Agree | Strongly Agree |
| Work itself | 1 | 2 | 3 | 4 | 5 |
| 1.Being able to keep busy all the time | 1 | 2 | 3 | 4 | 5 |
| 2.The chance to work alone on the job | 1 | 2 | 3 | 4 | 5 |
| 3.My work allows me to use my skills and abilities optimally | 1 | 2 | 3 | 4 | 5 |
| 4.I have opportunities to make independent decisions in my work | 1 | 2 | 3 | 4 | 5 |
| 5.My work gives me a feeling of accomplishment | 1 | 2 | 3 | 4 | 5 |
| 6.My work load is appropriate | 1 | 2 | 3 | 4 | 5 |
| 7.I like doing the things I do at work | 1 | 2 | 3 | 4 | 5 |
| Job return | 1 | 2 | 3 | 4 | 5 |
| 8.I am satisfied with my salary | 1 | 2 | 3 | 4 | 5 |
| 9.The income I receive in my unit is the same as those in other organizations | 1 | 2 | 3 | 4 | 5 |
| 10.There is the balance between remuneration and workload | 1 | 2 | 3 | 4 | 5 |
| 11.I am satisfied with the welfare policy and social insurance benefits | 1 | 2 | 3 | 4 | 5 |
| 12.I am satisfied with the distribution of wages and bonuses as well as the reward and punishment scheme | 1 | 2 | 3 | 4 | 5 |
| 13.I am satisfied with the praise and reward | 1 | 2 | 3 | 4 | 5 |
| Working conditions | 1 | 2 | 3 | 4 | 5 |
| 14.I am satisfied with the medical equipment | 1 | 2 | 3 | 4 | 5 |
| 15.I am satisfied with the drug allocation | 1 | 2 | 3 | 4 | 5 |
| 16.I am satisfied with the number of healthcare workers | 1 | 2 | 3 | 4 | 5 |
| 17.I am satisfied with the working environment | 1 | 2 | 3 | 4 | 5 |
| 18.I am satisfied with the working facilities | 1 | 2 | 3 | 4 | 5 |
| Professional growth | 1 | 2 | 3 | 4 | 5 |
| 19.There are many opportunities for promotion | 1 | 2 | 3 | 4 | 5 |
| 20.The opportunity for promotion in my unit is the same as those in other organizations | 1 | 2 | 3 | 4 | 5 |
| 21.There are many opportunities for training | 1 | 2 | 3 | 4 | 5 |
| 22.There are many opportunities to update professional skills and knowledge | 1 | 2 | 3 | 4 | 5 |
| 23.I have a good career prospect | 1 | 2 | 3 | 4 | 5 |
| Interpersonal relationships | 1 | 2 | 3 | 4 | 5 |
| 24.I get along well with my colleagues | 1 | 2 | 3 | 4 | 5 |
| 25.I have to work harder because of the incompetence of my colleagues | 1 | 2 | 3 | 4 | 5 |
| 26.I am satisfied with the interpersonal communication | 1 | 2 | 3 | 4 | 5 |

| Table S2 Description of variables assessing job satisfaction | |
| --- | --- |
| Variable Name | Description |
| Work itself | The definition of the work itself is how the employee perceives their current work. |
| Job return | Job return refers to the employees’ perception about their work remuneration and work accomplishment. |
| Working conditions | Working conditions refers to working facilities, the working system, and the working environment. |
| Professional growth | Professional growth refers to receiving studies and tests in the execution of professional roles to seek for progress and growth of professional competence. |
| Interpersonal relationships | Interpersonal relationships evaluate to what extent employees feel companionship and the support they want from others. |

Table S3 Field guide for the interview

| Interview participants | Questions |
| --- | --- |
| RTMSs | 1.How did you feel when you worked in the THCs?  2.Why did you leave your position? / Why did you stay in your position?  3.What were the problems that you have faced in the THCs?  4.What do you think of the career prospect of the rural GPs?  5.What can the government and THCs do to encourage you to stay in rural areas?  6.What do you think of the RTME programme?  7.What are your suggestions for the development and use of RTMSs? |
| Administrators of county health bureau | 1. How many RTMSs broke the contract and why did they break the contract in recent years?  2.What factors influenced RTMSs’ decisions to stay in their positions for a long time?  3.What can be done to encourage RTMSs to stay in rural areas?  4. What do you think of the RTME programme?  5.What are your suggestions for improving retention of the rural health workforce? |
| Administrators of THCs | 1. What do you think of the first group of the RTMSs?  2.What factors influenced RTMSs’ decisions to stay in their positions for a long time?  3.What can be done to encourage RTMSs to stay in rural areas?  4. What measures have you taken to encourage RTMSs?  5. What do you think of the RTME programme?  6.What are your suggestions for improving retention of the rural health workforce? |

**Confirmatory factor analysis results**

The initial 26-item five factor model indicated poor fit: RMSEA = 0.089, GFI = 0.752, AGFI = 0.699, CFI = 0.893, IFI = 0.895, TLI = 0.880. Modification indices suggested that there was a large error covariance between item 17 (The working environment in my unit) and item 18 (The workspace in my unit). And item 18 had lower factor loading compared to item 17. As a result, item 18 was removed. The fit of the model excluding item 18 had acceptable CFI, IFI, and TLI values, though RMSEA, GFI, and AGFI values were not acceptable. Item 7 (My workload is appropriate) and item 20 (The opportunity for promotion in my unit is the same as those in other hospitals) were eliminated because of non-significant loadings on their respective constructs. After the removal of three items, RMSEA, CFI, IFI, and TLI values were each acceptable, whereas GFI and AGFI values were not acceptable. Then item 2 (The chance to work alone on the job), item 3 (My work allows me to use my skills and abilities optimally), item 1 (Being able to keep busy all the time), item 19 (The opportunity for promotion), item 10 (The balance between remuneration and workload), item 5 (My work gives me a feeling of personal accomplishment) and item 22 (I can learn more professional knowledge and social knowledge in my work) were removed according to the modification indices. After removing 10 items, all indices met the reference value, and the final 16-item, 5-factor model exhibited very good fit (χ²/df =1.218, RMSEA =0.041, GFI=0.902, AGFI=0.859, CFI=0.985, IFI=0.985, TLI=0.981) (see Table S1).

Table S4 Results of confirmatory factor analyses of the job satisfaction scale

| χ²/df | RMSEA | GFI | AGFI | CFI | IFI | TLI |
| --- | --- | --- | --- | --- | --- | --- |
| 1.218 | 0.041 | 0.902 | 0.859 | 0.985 | 0.985 | 0.981 |


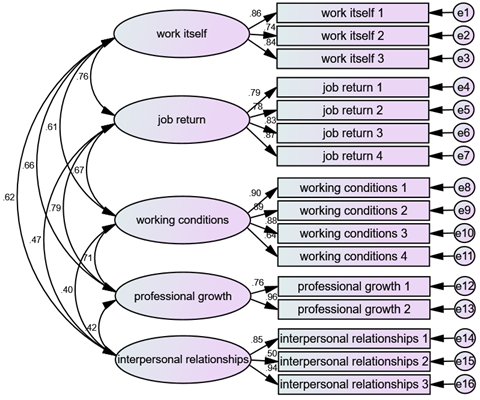


Fig S1 The final model and standardized model path
